# Novel Therapies in Takayasu Arteritis

**DOI:** 10.3389/fmed.2021.814075

**Published:** 2022-01-12

**Authors:** Francesca Regola, Martina Uzzo, Paola Toniati, Barbara Trezzi, Renato Alberto Sinico, Franco Franceschini

**Affiliations:** ^1^Department of Clinical and Experimental Sciences, University of Brescia, Brescia, Italy; ^2^Rheumatology and Clinical Immunology Unit, ASST-Spedali Civili of Brescia, Brescia, Italy; ^3^Department of Medicine and Surgery, University of Milano-Bicocca, Monza, Italy; ^4^Nephrology and Dialysis Unit, ASST-Monza, Ospedale San Gerardo, Monza, Italy

**Keywords:** Takayasu Arteritis, novel therapies, bDMARDs, biologics, heart

## Abstract

Takayasu Arteritis (TAK) is a large-vessel vasculitis that preferentially involves the aorta and its primary branches. Cardiac involvement is frequent in TAK and is a major determinant of the patient's outcome. Glucocorticoids (GC) are the mainstay of therapy for TAK, with high doses of GC effective to induce remission. However, relapses are common and lead to repeated and prolonged GC treatments with high risk of related adverse events. Potential GC toxicity is a major concern, especially because patients with TAK are young and need to be treated for several years, often for the whole life. Conventional immunosuppressive drugs are used in patients with severe manifestations but present some limitations. New therapeutic approaches are needed for patients with refractory disease or contraindications to conventional therapies. Fortunately, major progress has been made in understanding TAK pathogenesis, leading to the development of targeted biotherapies. In particular, IL-6 and TNF-α pathways seems to be the most promising therapeutic targets, with emerging data on Tocilizumab and TNF inhibitors. On the other hand, new insights on JAK-Inhibitors, Rituximab, Ustekinumab and Abatacept have been explored in recent studies. This review summarizes the emerging therapies used in TAK, focusing on the most recent studies on biologics and analyzing their efficacy and safety.

## Introduction

Takayasu Arteritis (TAK) is an idiopathic large-vessel vasculitis (LVV) that preferentially involves the aorta and its primary branches. It is usually considered to be most common in Asia, while in the USA and Europe is defined as a rare disease with an incidence of 1–3 per million people (1). It is most prevalent among females between the ages of 10–40 years (2). TAK is characterized by granulomatous inflammation of the aorta and large arteries wall, leading to stenosis, occlusion, dilatation and aneurysm formation. Main symptoms are consequences of vessels occlusion and reduced blood flow, like limb claudication, angina, hypertension secondary to renal arteries stenosis, lightheadedness or other neurologic symptoms due to cerebral arteries insufficiency. However, patients can also report arterial pain, like carotidynia, and non-specific constitutional symptoms, such as weight loss, low-grade fever and fatigue. In addition to these, TAK is often complicated by cardiovascular, cerebrovascular and renal morbidity.

## Cardiovascular Insights in Takayasu Arteritis

Among all kind of vasculitis, TAK is one of the diseases with the most frequent heart involvement. In TAK the whole aorta can be affected along its entire length, and all aortic branches can be involved. Thoracic and abdominal aorta are the most common affected vessels, but heart involvement has been demonstrated in up to a third of TAK patients. Cardiac manifestations can be various with coronary, valvular and myocardial involvement. They are not always clinically evident, especially in early phases, but are related to a poorer prognosis (3).

Acute myocardial infarction (MI) is rarely reported as a clinical manifestation of TAK but coronary lesions have been detected by coronary computed tomography angiography (CTA) in up to 53% of TAK patients (4). In particular, coronary stenosis is the most typical lesion found in TAK patients, and usually affects the coronary ostia and proximal vessel segments. Coronary aneurysms may also occur but are less frequent (5). Moreover, using myocardial scintigraphy and cardiac magnetic resonance imaging (MRI) has been demonstrated that 53% of TAK patients present myocardial perfusion abnormalities (6) and 27% of them present myocardial scarring (7), indicating previously unrecognized or chronic myocardial damage. However, <10% of patients became symptomatic for angina or MI (8, 9).

Besides coronary involvement, the most frequent cardiac manifestation of TAK are valvular abnormalities, found in more than 60% of patients (10). Aortic regurgitation is the most common type of valve disease and is associated with disease activity (11). Aortic insufficiency is considered to be related to aortic valves thickening or aortic root enlargement (12). Pulmonary, mitral and tricuspid regurgitations are less common and valvular stenosis is rare (13).

In patients with TAK other rarer cardiac manifestations have been reported, like myocarditis, pericarditis and pulmonary hypertension. More specifically, myocardial involvement has been reported in 6% of patients, usually presenting with mild insidious symptoms at onset, but leading to a later heart failure with poor prognosis (13). On the other hand, only few cases of pericarditis associated with TAK has been reported, usually as an initial manifestation of the disease (14) and only in a minority of patients a mild pulmonary hypertension has been observed (15).

Finally, the association between adverse cardiovascular events and glucocorticoids (GC) is a major concern that needs to be considered in the management of TAK patients. In fact, GC treatment contributes to the exacerbation of cardiovascular risk factors. GC administration increases blood pressure in a dose dependent fashion, mediated by both an increased peripheral vascular resistance and by a direct effect on mineralocorticoid receptor. GC treatment also increases the risk of glucose intolerance and diabetes, dyslipidaemia and central obesity. For these reasons, EULAR recommendations suggest screening all patients with TAK for treatment-related and cardiovascular comorbidities and recommend prophylaxis and life-style advice to reduce cardiovascular risk and treatment-related complications (16).

## Takayasu Arteritis: Conventional DMARDs

The mainstay of therapy for the induction of remission in TAK are systemic glucocorticoids (GC), with a commonly used initial prednisone dosage of 0.5–1 mg/Kg/day. EULAR recommendations published in 2018 suggest an initial dose of 40–60 mg/day for the majority of patients and, to date, there is no evidence that a higher starting dose improves the outcome (16). A high initial dose of GC is recommended also by the very recent 2021 ACR guidelines, due to the potential organ damage and life-threatening events associated with TAK onset. However, ACR guidelines allows to consider lower doses for patients with newly active, non-severe disease (e.g., patients with constitutional symptoms and without limb ischemia) (17).

However, although most patients initially achieve disease remission, relapses or disease progression are seen in more than half of patients during GC tapering (18). In addition, chronic GC therapy is associated with adverse effects, such as diabetes, hypertension, early cardiovascular disease, infections, osteoporosis and growth restriction in children.

Given the high frequency of GC adverse effects and the high rate of relapse during tapering, the upfront use of immunosuppressives in addition to GC seems to be the most preferable management strategy in TAK patients. Based on these considerations, EULAR recommendations and ACR guidelines advise an initial treatment with high-dose GC in combination with a GC-sparing agent in all TAK patients rather than GC alone (16, 17).

However, the choice of the immunosuppressive drug remains a challenge for several reasons. First of all, most of the evidence on their efficacy comes from observational studies with limited number of patients, especially for conventional synthetic disease modifying anti-rheumatic drugs (csDMARDs). Cohort studies have showed that methotrexate (45), azathioprine (46), mycophenolate mofetil (47), leflunomide (48) and cyclophosphamide (49) could improve clinical and radiological manifestations of TAK and have a GC-sparing effect. However, there are no randomized trials comparing the efficacy of different csDMARDs, and two recent meta-analysis on csDMARDs in TAK indicated similar efficacy rates between these drugs (25, 50). Therefore, clinical practice typically reflects the results of these low level of evidence data and expert opinion.

Secondly, TAK patients can be very different, and several individual factors need to be considered in order to choose the better treatment, including disease manifestations and severity, age, sex, comorbidities, contraindications, pregnancy plan, and also cost and availability of specific agents.

EULAR recommendations in 2018 suggested choosing as first line agent a csDMARD among methotrexate, azathioprine, mycophenolate mofetil or leflunomide. Switching from one csDMARD to another is considered a feasible option when a patient does not tolerate the first choice. On the other hand, cyclophosphamide is suggested to be used only if other treatments have failed or have not been tolerated, because of its high risk of long-term adverse events and infertility (16).

However, new therapeutic approaches are needed for patients with refractory disease or contraindications to conventional therapies. Fortunately, major progress has been made in understanding the pathogenesis of TAK, leading to the development of targeted biological disease modifying agents (bDMARDs). Tumor Necrosis Factor-α (TNF-α) and Interleukin 6 (IL-6) seem to be the most promising therapeutic targets, but other pathways have been studied, and will be discussed in the next sections (Table 1).

**Table 1:** New targeted therapies in Takayasu Arteritis.

**Drug**	**Pathogenetic basis**	**Evidence in TAK**	**Recommendations and** **clinical use**
**TNF-α inhibitors (infliximab, etanercept, adalimumab, golimumab, certolizumab pegol)**	**Inhibitors of TNF-α (bDMARDs):** • TNF-α has a major role in the development of TAK granulomatous inflammation • In active TAK higher serum levels of TNF-α and higher mRNA expression and intracellular production by T cells	• Cohort studies and open-label prospective study, showing positive results in TAK patients (clinical improvement, GC sparing effect, higher sustained remission rate compared to cDMARDs) (19–24) • No RCTs • Meta-analysis with 19 observational studies, showing more than 80% of treated patients attaining at least partial clinical response (25) • Good safety profile in cohort studies	• **2018 EULAR recommendations:** TNF-α inhibitors as second line treatment in TAK patients resistant to csDMARD • **2021 ACR guidelines:** TNF-α inhibitors as first line treatment, like methotrexate and azathioprine
**Tocilizumab**	**Anti-IL-6r (bDMARD)** • IL-6 is a pro-inflammatory cytokine • Higher IL-6 levels in TAK patients compared to HC and in TAK patients with active disease compared to patients with low disease activity	• Cohort studies, showing positive results in TAK patients (clinical improvement, GC sparing effect, higher sustained remission rate compared to cDMARDs) (22, 26–30) • Meta-analysis with 22 observational studies, showing more than 87% of treated patients attaining at least partial clinical response (25) • One RCT (TAKT study): relapse-free survival tended to be improved in treated patients, but no statistical significance. Longer-term open-label extension showed GC sparing effect, lower radiological disease progression, better PROs (31, 32) • Good safety profile in cohort studies and RCT	• **2018 EULAR recommendations:** tocilizumab as second line treatment in TAK patients resistant to csDMARD • **2021 ACR guidelines:** tocilizumab as second line treatment in patients with inadequate response to other immunosuppressive therapies
**JAK-Inhibitors (tofacitinib, upadacitinib)**	**Inhibitors of JAK-STAT signaling pathway (tsDMARDs)** • block signaling of cytokine implicated in TAK pathogenesis (type 1 and 2 interferons, IL-6, IL-12, IL-17 and IL-23) • suppress tissue-resident memory T cells and reduce inflammatory-related vascular damage	**Tofacitinib:** • Case reports and one prospective observational study, showing positive results in TAK patients (clinical improvement, lower radiological disease progression, superior to methotrexate) (33–37) • Good safety profile • One ongoing RCT (NCT04299971) **Upadacitinib:** • One ongoing RCT (NCT04161898) **No data on other JAK-Inhibitors**	• Only case reports • Not included in 2018 EULAR recommendations or 2021 ACR guidelines
**Rituximab**	**Anti-CD20 (bDMARD)** • In TAK patients B-cells infiltrates in the inflamed arteries adventitia and high levels of activated B-cell subsets in the peripheral blood • Rituximab blocks B cell differentiation and B-T cell stimulation	• Isolated case reports on rituximab in TAK with contradictory results (38–42) • No RCTs, no meta-analysis, no ongoing trial	• Very limited evidence with contradictory results • Only case reports • Not included in 2018 EULAR recommendations or 2021 ACR guidelines
**Abatacept**	**Soluble fusion protein CTLA4-Ig (bDMARD)** • In TAK patients B-cells infiltrates in the inflamed arteries adventitia and high levels of activated B-cell subsets in the peripheral blood • Abatacept blocks B-T cell co-stimulation	• One RCT with 34 TAK patients: abatacept not associated with a longer median duration of remission compared to placebo (43)	• **2021 ACR guidelines**: Abatacept is not recommended in TAK
**Ustekinumab**	**Anti-p40. IL-12 and IL-23 inhibitor (bDMARD)** • Th17 and Th1 pathways contribute to TAK pathogenesis • Ustekinumab target p40, a common subunit of IL-12 and IL-23 (main cytokines involved in Th17 and Th1 pathways)	• A small prospective observational study (improvement in clinical symptoms but no changes in intramural enhancement on MRA) (44) • One ongoing RCT (NCT04882072)	• Very limited evidence • Not included in 2018 EULAR recommendations or 2021 ACR guidelines

*TNF, Tumor necrosis factor; GC, glucocorticoids; cDMARDs, conventional disease modifying agents; bDMARDs, biological disease modifying agents; tsDMARDs, targeted synthetic disease modifying agents; IL, interleukin; IL-6r, interleukin-6 receptor; HC, healthy controls; MRA, magnetic resonance angiography; RCT, randomized controlled trial*.

## Takayasu Arteritis: New Targeted Therapies

### TNF-α Inhibitors (TNFi)

Tumor necrosis factor α (TNF-α) plays a major role in the development of granulomatous inflammation that is typical of TAK. Moreover, TAK patients with an active disease showed higher serum levels of TNF-α and higher mRNA expression and intracellular production by T cells if compared to inactive TAK patients (51, 52).

Currently, five TNF-α inhibitors (TNFi) are approved for rheumatic diseases by FDA and EMA: infliximab, etanercept, adalimumab, golimumab, and certolizumab pegol. Adalimumab and golimumab are fully human IgG1 antibodies, infliximab is a chimeric IgG1 antibody, etanercept is a fusion protein comprised of a human IgG1 Fc portion and the p75 TNF receptor, and certolizumab pegol is a PEGylated Fab fragment of a humanized anti-TNF antibody.

Several cohort studies on the successful use of different TNFi have been reported in patients with TAK but to date no RCTs have been published. A French multicenter open-label prospective study published in 2020, described the benefit-risk ratio of infliximab in TAK patients with refractory disease to conventional therapy. Between 2014 and 2017, 23 patients were treated with infliximab and a clinical improvement was observed in 64% of patients after a median treatment duration of 36.9 months. The median GC dose was reduced by 50% and no safety concerns were raised by the study, with only few reported adverse event during the 3 years of follow-up (19).

A recent meta-analysis by Misra et al. (25) analyzed 19 observational studies on TNFi in TAK, showing that more than 80% of treated patients attained at least partial clinical response, angiographic stabilization, improvement in PET-CT and normalization of inflammatory markers. Relapse rate was estimated as 32% but with considerable heterogeneity across studies. TNFi showed also a GC-sparing effect.

Similar results were reported in a previous review published in 2014 and including 120 TAK patients with active disease and treated with anti TNFi in 20 observational studies: 109 patients received infliximab, 17 etanercept and 9 adalimumab. Remission was achieved in 70–90% of cases after TNFi treatment and 40% of patients stopped glucocorticoids (20).

More specifically, a population-based cohort study from Norway included 78 TAK patients, comparing patients treated with TNFi and with csDMARDs. Patients treated with TNFi had a higher sustained remission rate and a lower risk of new lesion development if compared to patients on csDMARDs (42 vs. 20% and 10 vs. 40%, respectively) (21). Similar results were reported in 2015 by Mekinian et al. (22) on behalf of the French Takayasu Network.

All together, these data support the use of TNFi in TAK. Notably, the great majority of these patients received infliximab (19), while the experience with etanercept, adalimumab and golimumab is more limited (22, 23). Only one case series on certolizumab pegol has been published. In this report 10 female patients with TAK were treated with certolizumab pegol, achieving remission in all cases. Interesting, due to its safety during pregnancy, certolizumab pegol could present a specific advantage in TAK patients who are frequently female and young (24).

### Tocilizumab

IL-6 is a pro-inflammatory cytokine, responsible for stimulating acute phase protein synthesis and for neutrophils production and B- and T- cells activation. Several studies had suggested that IL-6 plays a crucial role in TAK pathogenesis (53, 54). Higher levels of IL-6 have been demonstrated in TAK patients compared to healthy controls and in TAK patients with active disease compared to patients with low disease activity (51). Tocilizumab is a humanized monoclonal antibody blocker of IL-6 signaling and has been approved for the treatment of Giant Cells Arteritis. The clinical efficacy of tocilizumab in TAK was reported for the first time in 2008 (26), followed by several cohort studies published in the subsequent years (22, 27, 28).

In 2018 the French Takayasu network published a retrospective multicenter study on 46 TAK patients treated with tocilizumab. Under tocilizumab treatment, a significant decrease in the median NIH scale and in the daily prednisone dose was observed. Moreover, the event-free survival was significantly better in patients treated with tocilizumab compared to cDMARDs (29).

The above-mentioned meta-analysis performed by Misra et al. (25) included 22 observational studies on tocilizumab in TAK patients. Pooling data from these studies, authors described tocilizumab effective in attaining at least a partial clinical response in 87% patients although the results were heterogenous among studies. Tocilizumab demonstrated also to induce angiographic stabilization, PET-CT improvement and median prednisolone dose reduction. In a previous review 70 cases of TAK patients with relapsing or refractory disease and treated with tocilizumab were reported. Overall, 80% of patients showed a clinical and laboratory improvement after 3 months of therapy and <20% of patients had a relapse during the treatment period (30).

Besides observational studies, a phase 3 RCT was published in 2018: the TAKT study. Thirty-six patients with relapsing TAK were randomized to receive tocilizumab 162 mg subcutaneous weekly or placebo. The primary endpoint was the time to occurrence of the first relapse, as defined by Kerr's criteria, but it was not met. In fact, after 1 year of follow-up, relapse-free survival tended to be improved in patients treated with tocilizumab, but the results did not reach statistical significance [HR 0.41 (95.41% CI 0.15 to 1.10; *p* = 0.0596)] (31). However, this study was felt to be underpowered (36 participants). Recently, the longer-term open-label extension of this trial was published, with patients in both arms treated with tocilizumab until 96 weeks. Endpoints of the extension analysis were steroid-sparing effects of tocilizumab, radiological disease progression, patient-reported outcomes and safety. The median glucocorticoid dose was significantly reduced from baseline to week 96, with 46.4% of patients reducing their prednisolone dose below 0.1 mg/kg/day. Most patients presented an improvement (17.9%) or a stabilization (67.9%) on imaging evaluations after 96 weeks compared to baseline, with only 4 patients showing a progression of vascular involvement. Mean SF-36 mental component summary scores improved rapidly by week 12 and 24 of tocilizumab treatment and improvement was maintained till week 96. The most frequently recorded adverse effects in the trial were infections but the long-term safety of tocilizumab in patients with TAK was consistent with the known safety profile of this drug in Rheumatoid Arthritis (32).

### JAK-Inhibitors

JAK-Inhibitors are a more recent family of drugs, classified as targeted synthetic DMARDs (tsDMARDs). They inhibit the activity of one or more Janus kinase enzymes (JAK1, JAK2, JAK3, TYK2), interfering with the JAK-STAT signaling pathway and thereby blocking cytokine signaling. In particular, JAK inhibition suppresses the production of type 1 and 2 interferons and many cytokines including IL-6, IL-12, IL-17, and IL-23, which are implicated in TAK pathogenesis (55, 56). It has also been demonstrated that JAK1 and JAK3 signaling is important in chronic inflammation of large arteries and that JAK inhibition can suppress tissue-resident memory T cells and reduce inflammatory-related vascular damage (57).

Tofacitinib (TOF) is a JAK3 and JAK1 inhibitor and it has been studied in TAK patients in the last 2 years (33–35). A recent observational study on 5 patients reported its efficacy in TAK, with 4 patients out of 5 achieving clinical response. Moreover, three of these patients presented an improvement and a stabilizations of artery stenosis and mural thickness in vascular Doppler (36). Recently, a study comparing TOF and methotrexate in TAK patients has been published in China. TOF demonstrated to be superior to methotrexate for complete remission with a tendency to prevent relapse and tapering GC. A good safety profile for TOF was also documented in these patients (37).

More information on tofacitinib efficacy in TAK patients will be provided by an ongoing trail (ClinicalTrials.gov Identifier NCT04299971). At the same time, another JAK1 inhibitor is subject of a clinical trial: upadacitinib. In fact, a phase-3, multicenter, placebo-controlled study (SELECT-Takayasu) is now recruiting (ClinicalTrials.gov Identifier NCT04161898).

### Rituximab

There is increasing evidence about the possible role of B-cells in the pathogenesis of TAK. B-cells infiltrates in the inflamed adventitia of affected arteries and high levels of activated B-cell subsets, particularly plasmablasts, in the peripheral blood of TAK patients have been described (39, 58). These findings suggest a potential role for B-cell depleting therapy in TAK. Rituximab is a chimeric anti-CD20 monoclonal antibody that induces a depletion of B-cells.

Isolated case reports on the use of rituximab reported favorable outcomes, but are limited by reporting bias (38–40). To date, only two retrospective case series of TAK patients treated with rituximab have been reported. *Pazzola et al*. described seven patients with refractory disease treated with rituximab. Despite treatment, four patients had evidence of persistent disease activity and/or radiographic disease progression during follow-up. Only three out of seven patients achieved complete remission (41). On the contrary, *Nakagomi et al*. described eight TAK patients treated with rituximab, with all but one with a clinical response after treatment (42).

In conclusion, data on the efficacy of rituximab in TAK are very heterogenous. Further studies would be necessary to understand rituximab role in TAK treatment.

### Abatacept

As above mentioned, in TAK pathogenesis a possible role of B-cells has been theorized. At the same time, B-cells activation need costimulatory signals by activated T lymphocytes, macrophages, and dendritic cells. Abatacept is a soluble fusion protein comprising CTLA-4 and the Fc portion of immunoglobulin G1 (CTLA4-Ig). This drug prevents CD80/CD86 from binding to CD28 on the surface of the T-cells, resulting in failure of the costimulatory signal required for T-cell activation.

A randomized controlled trial enrolling 34 TAK patients has been conducted to test the efficacy of abatacept to prevent disease relapse (43). The primary end point of the study was not met, with abatacept not associated with a longer median duration of remission compared to placebo (5.5 vs. 5.7 months, p: ns). Moreover, the relapse-free survival rate at 12 months was 22% for patients receiving abatacept and 40% for those receiving placebo. To date, this study does not support the use of abatacept in TAK.

### Ustekinumab

The Th17 and Th1 pathways contribute to the systemic and vascular manifestations of TAK (53). IL-12 and IL-23 are two key cytokines involved in Th1 and Th17 polarizations, respectively, and IL-12B gene region has been identified as a susceptibility gene for TAK (59). These findings suggest that IL-12 and IL-23 are implicated in the pathogenesis of TAK. These two cytokines share a common subunit (p 40), which is target by ustekinumab, a humanized anti-p 40 monoclonal antibody.

In a small prospective observational study, three patients with active TAK were treated with ustekinumab in association with csDMARDs and glucocorticoids. After ustekinumab, all three patients presented an improvement in clinical symptoms and a decrease in inflammation markers, but no changes in intramural enhancement on magnetic resonance angiography (MRA) were achieved (44).

These results are very interesting but still preliminary. Further information on the efficacy of ustekinumab in TAK will be provided by a proposed phase-3, multicenter, placebo-controlled study (ClinicalTrials.gov Identifier NCT04882072).

## Surgical Management

Thanks to all these new therapeutic opportunities, most patients achieve remission and do not develop irreversible vascular damage. However, it is possible that the diagnosis occurs at a stage when stenotic or occlusive lesions have already occurred. Such lesions might be not reversible by medical treatment and, if they are hemodynamically significant, may require revascularization. Most common examples are represented by cerebrovascular disease due to carotid or vertebral stenosis, coronary artery disease, severe coarctation of the aorta, renovascular hypertension or limb claudication. Such interventions need to be considered when vascular lesions are symptomatic and only if refractory to medical management, which represents the first-choice treatment (17). Not only stenotic, but also aneurismatic complication can occur in TAK patients, and surgical management can be necessary in patients with progressive aneurysm enlargement with high risk of rupture or dissection (16).

EULAR, ACR, ESC (European Society of Cardiology) and ESVS (European Society for Vascular Surgery) guidelines recommend performing elective endovascular interventions or reconstructive surgery during stable remission (16, 17, 60, 61). Surgical interventions in patients with active disease are associated with an increased risk of complications and with higher risk of requiring revision for relapse or progression of symptomatic disease (62, 63).

The method of choice for vascular interventions in patients with TAK depends on the anatomic location of the vascular damage, timing, disease activity and other factors, and should a collaborative decision between vascular surgeons and rheumatologists (16, 17). With recent advances in endovascular treatment, the use of percutaneous endoluminal angioplasty has progressively increased in TAK patients. Endovascular management is considered a feasible option especially for stenotic lesions, like in supra-aortic, iliac, and renal arteries stenosis (64–66). On the other hand, for inflammatory thoracic aortic aneurysms, open surgery with resection and replacement of the inflammatory aorta still represents the first-line standard treatment. However, successful outcomes after thoracic endovascular aortic repair (TEVAR) have been recently reported, showing that in the future TEVAR could represent a less invasive alternative in selected patients (67).

## Conclusions

TAK is a chronic disease, typically affecting young patients and associated with potential organ damage and life-threatening events. It requires a prompt and aggressive treatment with immunosuppressants to avoid irreversible complications. GC have been considered the mainstay in the treatment of TAK but they are characterized by high incidence of side effects and relapse during tapering. Alternative therapies with cDMARDs showed partial efficacy, with half of the patients experiencing relapses.

As discussed above, new therapeutic approaches with bDMARDs and tsDMARDs have showed promising results, with high efficacy and acceptable safety profile.

In 2018, based on these new insights, EULAR recommendations advised the use of bDMARDs in TAK. In particular, TNFi and Tocilizumab were suggested to be used as second line agents in patients with relapsing or refractory disease despite treatment with csDMARDs or in patients with contraindications to csDMARDs (16, 68).

The most recent ACR guidelines, published in 2021, suggest a similar but different approach. Also in this case, non-glucocorticoid immunosuppressive agents plus GC are recommended over GC monotherapy in all patients with TAK to minimize GC-related toxicity. However, ACR guidelines specifically referred to methotrexate, azathioprine and TNFi as first line therapies. Notably, among bDMARDs the panel specified favoring TNFi use over tocilizumab, even if the latest is suggested to be considered, especially when TNFi are contraindicated (17).

In conclusion, biological therapies can provide additional benefits to TAK patients, and they are gradually becoming part of the clinical practice. Nevertheless, there is still a need for high-quality studies, especially RCTs, to guide the management of TAK. Hopefully, the results of the above-mentioned ongoing trials will help to better treat this challenging disease in the future.

## Author Contributions

FF and RS conceived the study. FR and MU conducted a review of the literature and drafted the manuscript. FR, MU, BT, PT, FF, and RS reviewed and edited the manuscript and supported the study. All authors checked the final version of the manuscript.

## Conflict of Interest

RS has received consulting fees from GSK, Roche and Otzuka. The remaining authors declare that the research was conducted in the absence of any commercial or financial relationships that could be construed as a potential conflict of interest.

## Publisher's Note

All claims expressed in this article are solely those of the authors and do not necessarily represent those of their affiliated organizations, or those of the publisher, the editors and the reviewers. Any product that may be evaluated in this article, or claim that may be made by its manufacturer, is not guaranteed or endorsed by the publisher.
